# Changes in Serum Lipids and Blood Glucose in Non Diabetic Patients with Metabolic Syndrome after Mixed Meals of Different Composition

**DOI:** 10.1155/2012/215052

**Published:** 2012-02-01

**Authors:** Adriana Branchi, Adriana Torri, Cristina Berra, Emanuela Colombo, Domenico Sommariva

**Affiliations:** ^1^Centro per lo Studio e la Prevenzione dell'Aterosclerosi, Department of Internal Medicine, Fondazione IRCCS Cà Granda, Ospedale Maggiore Policlinico, University of Milan, via F. Sforza 35, 20122 Milan, Italy; ^2^Department of Internal Medicine 1, G. Salvini Hospital, Garbagnate Milanese, Milan, Italy

## Abstract

*Aims*. To investigate the postprandial changes in serum lipoproteins and blood glucose and to verify whether different nutrient composition of the meal elicits different response in patients with (MetS+) and without (MetS−) metabolic syndrome. *Research Design and Methods*. 50 MetS+ patients and 50 age- and sex-matched MetS− consumed a regular lunch chosen among those more similar to their usual diet. Blood was drawn in the morning after 12-hour fasting and 2 and 4:30 hours after the meal. *Results*. Serum triglycerides increased more in MetS+ (35%, 4:30 hours after the meal) than in MetS− (29%), HDL-cholesterol decreased 2 hours after the meal in both groups (−4% and −5%, resp.). Blood sugar similarly increased in both groups (19%, 2 hours after the meal in MetS+ and 17% in MetS−) and plasma insulin increased more and remained high longer in MetS+ (73.5 and 52.3 **μ**U/mL, 2 and 4:30 hours after the meal) than in MetS− (46.7 and 21.6 **μ**U/mL). Difference in nutrient composition of the meal (carbohydrate 57%, fat 28% versus carbohydrate 45%, fat 35%) was not associated with differences in postprandial levels of triglycerides, HDL-cholesterol, glucose, and insulin within each group. *Conclusions*. As compared with MetS−, MetS+ patients show a greater hypertriglyceridemic and hyperinsulinemic response to a regular lunch whatever the carbohydrate or fat content of the meal.

## 1. Introduction

Components of the metabolic syndrome (MetS), blood sugar (BG), serum triglycerides (TGs), and HDL cholesterol (HDL-C) undergo major changes during the day as a consequence of repeated meals. Several factors have been demonstrated to influence postprandial changes of glucose and lipid variables, among them fasting levels [1–5] and insulin sensitivity [1, 3, 6–8]. Fasting levels of BG and TG are higher, insulin sensitivity is lower than normal in MetS, and therefore both BG and TG are expected to increase more in patients with MetS than in those without MetS. The magnitude and duration of the increase is however related to the nutrient composition of the meal [5]. Since humans are at most part of the day in postprandial state, the effects of diet in the short term must be taken into account, especially because a large body of evidence shows that changes in postprandial lipoprotein pattern and BG are associated with changes in cardiovascular risk [9–14]. It is then conceivable that the evaluation of the effects of modified diets on BG and lipoprotein profile should include the postprandial state.

 Dietary recommendations [15] for the management of patients with the MetS emphasize reduction of total fat intake and its replacement with carbohydrate (CHO). Low-fat diet means lower postprandial lipemia and decrease of low-density lipoprotein cholesterol level, that is the primary target of therapy of MetS [15]. Concerns about this approach have been raised on the basis of the fact that diets low in fat are high in CHO that may have detrimental effects on BG, TG, and HDL-C. Some authors suggested that low-fat diets should be avoided in the treatment of the MetS and advocated lower intake of CHO [16]. Low-CHO diets improve insulin sensitivity, lower serum TG, and raise HDL-C [17, 18]. Diets with very low-CHO content have been shown to reduce the TG response to a fat load in normal weight men and women [19–21], but not in overweight women [22]. As compared with low-CHO diets, higher-CHO diets have been reported to be associated with a greater postprandial elevation of serum TG in diabetic [23] and nondiabetic patients [24, 25]. Low-fat diet appears then to have unfavorable effects on postprandial lipoprotein profile as well as on blood glucose and plasma insulin [16], and this may have particular implications in patients with insulin resistance who have an exaggerated TG and glycemic response to the meal [7]. However, in the above mentioned studies meals had extremely high or extremely low proportions of fat and CHO. The effects of meals with less extreme variations in macronutrients and that are more suitable for long-term dietary treatment of patients with the MetS are poorly known.

 We studied a group of nondiabetic patients with (MetS+) and without MetS (MetS−) in the morning before breakfast and 2 and 4:30 hours after lunch. The main purpose of the study was to investigate the changes in serum lipoprotein pattern and BG after a regular meal and to verify whether moderately different nutrient composition of the lunch elicits different response in MetS+ and MetS− patients.

## 2. Patients and Methods

### 2.1. Patients

The study was carried out on 50 patients (25 males and 25 females, 31 to 65 years) with MetS as diagnosed according to the criteria of the National Cholesterol Education Program Adult Treatment Panel III (NCEP ATPIII) [18] and on 50- age and sex-matched patients without MetS. The patients were selected among patients hospitalized for minor illnesses in the Department of Internal Medicine of the G. Salvini Hospital in Garbagnate Milanese. All the patients were in good clinical and nutritional conditions. Pathological history of patients included chest discomfort of noncardiac origin (21), hypertension (19), paroxysmal supraventricular arrhythmias (17), syncope (14), abdominal pain (10), osteoarthritis (6), dizziness (5), anxiety attacks (5), urticaria (2), and transient global amnesia (1). The exclusion criteria were a history of thyroid disease, diabetes mellitus, hepatic and renal disfunction, neoplastic disease, current pregnancy, unstable medical condition, and the current use of medications known to affect weight, appetite, blood lipids, and glucose. One male and 3 females (3 MetS+ and 1 MetS−) were hypercholesterolemic (274, 303, 307 and 310 mg/dL) and 5 males and 2 females MetS+ were hypertriglyceridemic (246, 247, 262, 288, 298, 314, and 380 mg/dL). All patients gave their informed consent to the study protocol, which was conducted according to the guidelines of the Declaration of Helsinki and had been approved by the local Ethic Committee.

### 2.2. Study Design

The main purpose of the study was to evaluate the metabolic effects of meals in conditions as close as possible to everyday life. The patients followed their usual diet and the test meal was chosen among those that most resembled their habitual diet. All meals were prepared by the hospital kitchen. The patients did not consume alcoholic beverages. The patients had their breakfast (12% of daily calories: carbohydrates 71%, protein 18%, fat 11% of total energy) at 8: 00 AM and their lunch (on average 1121±17 calories, 51% carbohydrates, 18% protein, 31% fat) at 12:30 PM. The composition of meals was assessed by an accurate nutritional analysis that was made for each subject by two of us (C. Berra and E. Colombo). Quantities of intake were estimated according to a table with standardized portion sizes (Atlante Ragionato di Alimentazione, Istituto Scotti Bassani, Milan, Italy).

### 2.3. Analytical Methods

Blood samples were collected in the morning at 8:00 after an overnight fast and before the breakfast, at 2:30 PM and at 5:00 PM, and immediately centrifuged. Total cholesterol was measured by CHOD-PAP method, serum TG by GPO-PAP method, HDL-C by HDL-C plus method, and BG by GOD-PAP method (Roche Diagnostics GmbH, Mannheim, Germany). Measurements of serum lipids, HDL-C, and BG were done on the Hitachi 917 autoanalyzer (Boehringer Mannheim, Germany). Apolipoprotein (apo) B and A-I were determined by immuno-turbidimetric method (Roche Diagnostics GmbH, Mannheim, Germany) and plasma insulin by radioimmunoassay (Insulin RIA, Adaltis Italia S.p.A., Casalecchio sul Reno, Italy). The accuracy of determinations was assessed according to the Intra- and Inter-laboratory Quality Control Program UNITY (Bio-Rad Laboratories S.r.l., Segrate, Italy). Insulin resistance (HOMA_IR_) was calculated according to Matthews et al. [26].

Body mass index (BMI) was calculated by dividing weight (in Kg) for height^2^ (in m.). Waist circumference was measured at the level of the umbilicus and hip circumference at the level of the greater trochanters. The magnitude of postprandial changes in serum lipids, lipoproteins, BG, and insulin was estimated by calculating the incremental area under the curve (IAUC), according to the trapezoidal method after subtraction of fasting values.

### 2.4. Statistical Analysis

Data are presented as mean ± SEM. Differences between groups were analyzed with the Student's *t*-test for unpaired data. Student's *t*-test for paired data was used to compare data within groups. The two tailed significance threshold was set at *P* < 0.05.

## 3. Results

### 3.1. Baseline

Table 1 summarizes physical characteristics and fasting metabolic variables of the 100 patients included in the study. With respect to MetS−, MetS+ patients were more obese, had greater waist to hip ratio, and had higher baseline serum TG, apo B, Apo A-I/HDL-C ratio, BG, plasma insulin, and HOMA_IR_ and lower HDL-C and apo A-I.

### 3.2. Postprandial Changes

**Figure 1:**
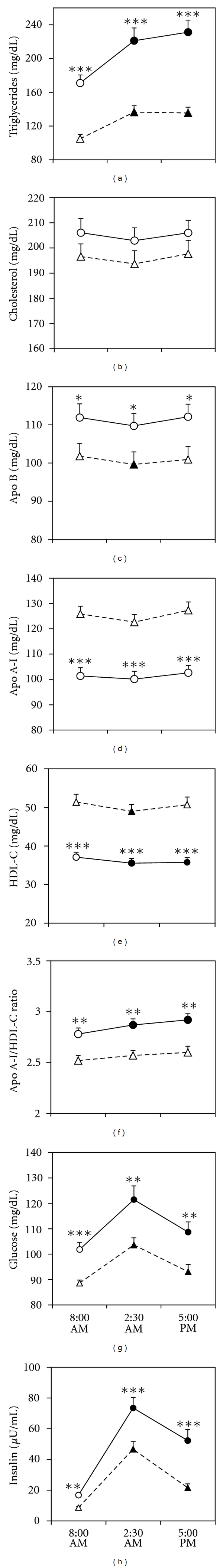
Line plots show the postprandial responses of serum lipids, HDL cholesterol, apolipoprotein A-I and B, blood glucose, and insulin in patients with MetS (–*⬤*–) and without MetS (- -▲- -). Vertical bars are the standard error of the mean. Filled symbols represent significant difference from baseline. *P < 0.05, **P < 0.01, ***P < 0.001 versus patients without MetS.

As shown in Figure 1, after the lunch serum TG significantly increased in both MetS+ (29% at 2:30 PM and 35% at 5:00 PM) and MetS− patients (30% and 29%, resp.). Despite the apparent similarity of postprandial increase of TG in the 2 groups, calculation of IAUC demonstrated that the increase of TG was significantly greater in MetS+ than in MetS− (Table 2), possibly due to the longer duration of hypertriglyceridemia in MetS+ patients. In both MetS+ and MetS−, HDL-C significantly decreased at 2:30 PM (−4% and −5%, resp.) and remained significantly lower than baseline at 5:00 PM in MetS+ (−4%), but not in MetS− patients. Apo A-I did not change significantly, while Apo A-I/HDL-C ratio significantly increased only in MetS+ group (3% at 2:30 PM and 5% at 5:00 PM). BG significantly increased in both MetS+ (19% at 2:30 PM and 7% at 5:00 PM) and MetS− (17% and 5%, resp.), without differences between the 2 groups as shown by the lack of significant difference of IAUC (Table 2). Plasma insulin increased more (Table 2) and remained high longer in MetS+ than in MetS− patients (Figure 1). In neither group of patients serum cholesterol and apo B significantly changed after meal.

### 3.3. Low Fat and Low CHO Meal

**Figure 2:**
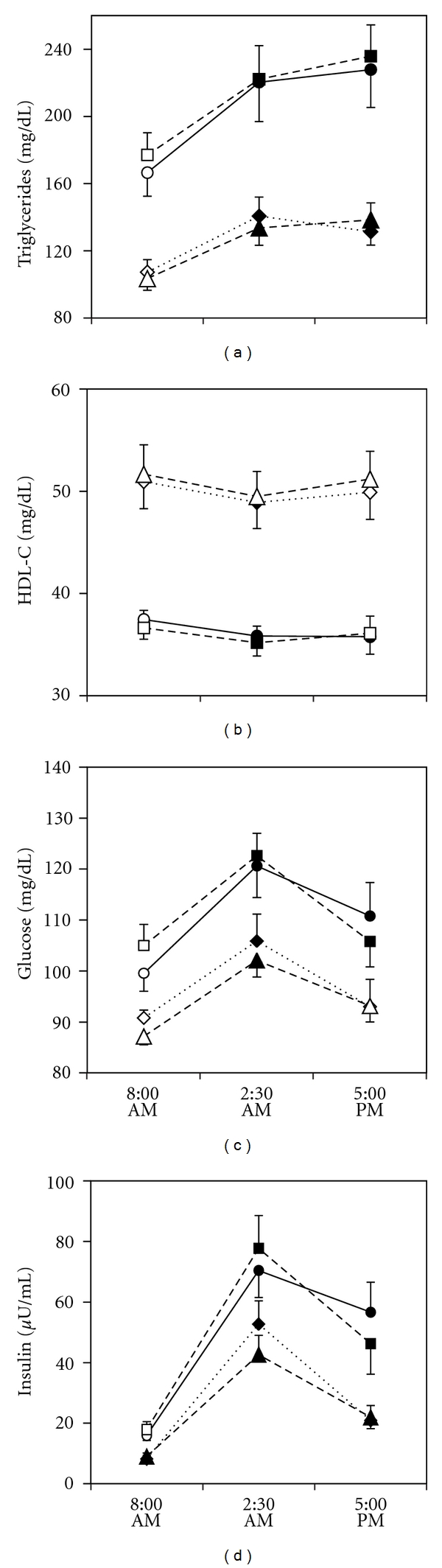
Line plots show the postprandial responses of serum triglycerides, HDL cholesterol, blood glucose, and insulin in patients on low-fat and on low-carbohydrate diet. (–*⬤*–) MetS+ low-CHO meal; (- -■- -) MetS+ low-fat meal; (- -*◆*- -) MetS− low-CHO meal; (- -▲- -) MetS− low-fat diet. Vertical bars are the standard error of the mean. Filled symbols represent significant difference from baseline.

On the basis of the median of CHO content, meals were subdivided into 2 groups: in the first one CHO accounted on the average for 45% of calories and fat 35%, and in the second one CHO accounted for 57% and fat 28% of calories (Table 3). Twenty nine MetS+ (14 males and 15 females) and 20 MetS− patients (11 males and 9 females) consumed a meal low in CHO; 21 MetS+ (11 males and 10 females) and 30 MetS− (14 males and 16 females) consumed a meal low in fat. As shown in Figure 2, the difference in nutrient composition of the meal was not associated with difference in postprandial levels of serum TG, HDL-C, BG, and plasma insulin within each group of MetS+ and MetS− patients.

## 4. Discussion

Nondiabetic MetS+ patients show a greater hypertriglyceridemic response to a meal than MetS− patients. This is the main conclusion of the present study and is in accordance with a series of previous observations that include insulin resistance [1, 3, 6–8], obesity [1, 2, 8, 25, 27], and fasting triglyceride level [1–4] among the determinants of postprandial lipemia. In MetS+ patients the increase of serum TG was associated with changes in HDL particles. The reduction of HDL-C was not coupled with a significant decrease of apo A-I, suggesting a remodeling of HDL particles, characterized by a loss of cholesterol, rather than a decrease of their concentration. The increase of apo A-I/HDL-C ratio further supports this hypothesis that on the other hand was already demonstrated by Dubois et al. [28] after different amounts of fat in the usual range of ingestion.

 As expected, in both MetS+ and MetS− patients, BG and insulin increased following the meal. In the fasted state BG and insulin were significantly higher in MetS+ than in MetS− patients and the difference persisted during the postprandial phase with the highest values reached 2 hours after the meal. The IAUC of glucose however was not significantly different between the 2 groups, whereas IAUC of insulin was significantly greater in MetS+ than that in MetS− patients. Hyperinsulinemia indicates insulin resistance that is the hallmark of the MetS [15] and was present in our series of MetS+ patients as shown by the high HOMA_IR_. Lack of a significant difference in IAUC of BG between MetS+ and MetS− patients in front of significantly greater IAUC of insulin suggests that hyperinsulinemia was sufficient to buffer in MetS+ patients the glycemic response to the meal.

 Khoury et al. [29] found that IAUC of glucose after high CHO (CHO 60%, protein 20%, and fat 20%) and high protein (CHO 30%, protein 50%, and fat 20%), but not after high fat (CHO 30%, protein 20% and fat 50%), liquid formula meals was significantly greater in 10 MetS patients than that in controls. The difference in IAUC of insulin reached the statistical significance only after the high-protein meal. Patients with MetS displayed higher postprandial TG than controls with all meals; the IAUC of TG was however significantly different only after high-fat and high-protein meals, but not after high-CHO meal. The highest values of IAUC of glucose in MetS patients were observed after the high-CHO meal and the highest IAUC of TG after the high-fat meal.

 In the present study, lipoprotein pattern and blood glucose did not have different response to the meal poor in CHO or in fat neither in MetS+ nor in MetS− patients. Our data are then at variance with the data of Khoury et al. [29] and with other studies showing that high-fat/low-CHO diets reduce lipid response to a meal as compared with low-fat/high-CHO diet [19–21, 23–25]. Several aspects must be taken into account in explaining the conflicting results, first of all the type of diets and the test meals. Studies on the effects of diet with different fat and CHO composition often used diets with extremely high or extremely low proportions of fat and CHO. Volek et al. [20] and Sharman et al. [21] in overweight and normal weight individuals observed that consumption of a very low-CHO diet (<10% of total energy) determined a lower TG response to an oral fat load than a diet low in fat (30% of energy). Compared with the low-fat diet, very low-CHO diet increased fasting LDL-C and HDL-C and decreased fasting TG and blood glucose. The effect of very low-CHO diet on fasting TG level is of considerable interest in the evaluation of postprandial lipemia since fasting TG level has been shown to be one of the most powerful predictor of postprandial lipemia in several different experimental conditions [1–4, 27] as well as in the present study. The lowering effect of very low-CHO diet on postprandial lipemia might then be explained, at least in part, by a decrease of fasting TG, rather than by an increase of the tolerance of a fat meal. Koutsari et al. [19] reported in a small group of normolipidemic men that after 3 days of low-CHO diet (18% of energy), TG and glucose response to a standard high-fat meal was lower than that observed after 3 days of low-fat diet (18% of total energy). Again, fasting TGs were significantly lower after the low-CHO than after the low-fat diet so the difference in basal TG may have influenced the incremental TG response to the meal. The authors however reported that total lipemic response to the fat meal was closely related to fasting TG concentration in patients following the low-fat diet but not in patients following the low-CHO diet.

 Less extreme diets also showed that low-CHO diet is associated with lesser increase in postprandial TG than low-fat diet. In an observational study on diurnal capillary TG profiles in free living patients eating regular meals, both absolute and incremental changes in TG concentrations during the day were significantly correlated with CHO intake [25]. In healthy volunteers [30] and in patients with type 2 diabetes [31], 2 weeks of low-CHO diet (40% CHO, 45% fat, and 15% protein) resulted in lower TG level throughout 8–12-hour period in response to breakfast and lunch than after 2 weeks of low-fat diet (60% CHO, 25% fat, and 15% protein). Fasting TG concentration was lower after low-CHO than after low-fat diet. In our MetS+ patients eating low-fat and low-CHO meal, fasting TG level was similar, and therefore we can rule out that the lack of a difference in fasting TG may have influenced postprandial response to the meal. The same is true for MetS− group.

Our low-fat and low-CHO meals had relative proportions of fat and CHO quite different, but not so different as in the above mentioned studies, and this might explain at least in part why we did not observe differences in postprandial BG and TG levels between the 2 groups of diet. Food constituents of the meals may further contribute to explain our findings, in particular, quality of foods containing CHO which were mainly of the low-glycemic-index type [32]. Harbis et al. [33] reported just in patients with MetS that test meals with comparable amounts of fat (28-29 g) and CHO (91–94 g), but with different glucose availability, differently affected lipid levels in postprandial state. TG significantly increased after meal rich in promptly absorbable CHO foods but not after meal containing slowly digestible CHO. Glycemia and insulinemia were significantly lower after the meal with low-glycemic-index than after meal with high-glycemic-index foods. The detrimental effects of low-fat/high-CHO meal do not seem to occur when the meal is largely based on fibre-rich, low glycemic index foods as in our case.

 Some limitations of our study should be noted. First, the patients were not on stable controlled diet. However, our purpose was not to study the long-term effects of diets on postprandial lipid and glucose metabolism but just to verify whether regular meals elicit different response in patients with and without MetS and whether different fat and CHO contents of the meal determined different postprandial response. It may be argued that 3 sample points do not allow an efficient analysis of postprandial lipemia and BG. Guerci et al. [34] and Carstensen et al. [3] found good correspondence between calculations of postprandial lipemia by 3-point sample analysis and 5–7 lipid determinations conventionally used in studies on postprandial lipemia. More recently, Weiss et al. [35] found that 4-hour lipemic response was highly predictive of 8-hour responses. In our study, the last sampling of serum lipids and BG was obtained after only 4:30 hours from the beginning of the lunch. Maximal response of both BG and TG occurs 2-3 hours after oral loads and the elevation of serum BG lasts for few hours whereas the elevation of TG is much longer. In a previous study on circadian triglyceridemia [36], we found that in healthy patients TGs in whole serum and in the density fraction <1.006 remained significantly higher than those in the fasted state from noon until the dinner at 7:30 PM and then increased again until midnight. In the present study, we have missed late values of postprandial serum TG. Therefore it is possible that we have underestimated duration, but not the magnitude of postprandial hypertriglyceridemia and the associated changes in HDL concentration.

In conclusion, our study demonstrates that MetS+ patients respond with exaggerated triglyceridemia and insulinemia to meals that closely reflect in percent terms the habitual diet in our population and that moderate low-fat/high-CHO and low-CHO/high fat meals are associated with similar changes in serum TG, HDL-C, blood glucose, and plasma insulin in the postprandial period. Both low-fat and low-CHO diets can then be recommended in patients with MetS without risk of undesirable effects on postprandial lipemia and glycemia, provided that meals are largely based on fiber-rich, low glycemic index foods. Long-term studies demonstrated that both diets improve fasting lipoprotein profile in patients with MetS and are effective in reducing the prevalence of the components of the MetS [37, 38]. In a 5-month study [39], we found that both low-CHO and low-fat diets, similar to meals of the present study, were effective in improving clinical and biochemical determinants of MetS; however low-CHO diet was associated with a greater decrease of serum TG and blood pressure, whereas only low-fat diet was associated with a decrease of LDL-cholesterol. Then, the choice between one or the other diet may depend on the specific presentation of the syndrome.

## Figures and Tables

**Table 1:** Physical characteristics and fasting metabolic profile of the patients included in the study.

Variables	With metabolic syndrome	Without metabolic syndrome	P*
	mean ± SEM	mean ± SEM	
Males/females	25/25	25/25	
Age (y)	55.3 ± 1.19	50.5 ± 1.21	N.S.
Body mass index (Kg/m^2^)	30.1 ± 0.71	26.2 ± 0.50	<0.001
Waist girth (cm)	100.5 ± 2.01	87.9 ± 1.60	<0.001
Waist/Hip ratio	0.94 ± 0.01	0.89 ± 0.01	<0.01
Serum triglycerides (mg/dL)	170.9 ± 9.59	105.0 ± 5.06	<0.001
HDL cholesterol (mg/dL)	37.1 ± 1.27	51.4 ± 2.01	<0.001
Apolipoprotein A-I (mg/dL)	101.4 ± 3.25	125.9 ± 3.04	<0.001
Apo A-I/HDL-C ratio	2.78 ± 0.06	2.52 ± 0.05	<0.002
Total cholesterol (mg/dL)	206.0 ± 5.63	196.6 ± 5.05	N.S.
Apolipoprotein B (mg/dL)	111.9 ± 3.61	101.8 ± 3.37	<0.05
Glucose (mg/dL)	101.8 ± 2.83	88.6 ± 1.17	<0.001
Insulin (*μ*U/mL)	16.8 ± 1.43	8.7 ± 0.85	<0.001
HOMA_IR_	4.29 ± 0.40	1.87 ± 0.16	<0.001

*Student's *t*-test for unpaired data.

**Table 2:** Incremental area under the curves (IAUCs) of serum triglycerides, HDL cholesterol, blood glucose, and insulin.

	Number	Triglycerides mg* 9 h/dL	HDL-C mg* 9 h/dL	Glucose mg* 9 h/dL	Insulin *μ*U* 9 h/mL
MetS+	50	301.3 ± 48.9*	−8.6 ± 2. 8	96.9 ± 22.3	299.7 ± 32.6^§^
MetS−	50	179.9 ± 28.5	−11.8 ± 5.6	73.0 ± 13.0	187.0 ± 23.0
MetS+ low CHO meal	29	319.3 ± 60.5	−9.4 ± 3.9	108.9 ± 32.4	296.1 ± 44.3
MetS+ low fat meal	21	276.4 ± 82.5	−7.4 ± 3.9	80.3 ± 28.9	304.6 ± 48.9
MetS− low CHO meal	20	180.5 ± 36.2	−10.5 ± 4.8	70.7 ± 24.9	216.3 ± 31.4
MetS− low fat meal	30	179.4 ± 41. 5	−12.6 ± 8.8	74.6 ± 14.4	167.5 ± 32.0

*P < 0.05; ^§^ *P* < 0.01 versus MetS−.

**Table 3:** Mean composition of the lunch of patients subdivided according to the median of CHO consumed.

Variables	Low CHO	Low fat	P*
Males/females	25/24	25/26	
Energy (Cal)	1114 ± 22.5	1138 ± 26.0	N.S.
Carbohydrate (%)	45 ± 0.6	57 ± 0.9	<0.001
Total fat (%)	35 ± 0.8	28 ± 0.9	<0.001
Saturated fat (%)	10 ± 0.5	9 ± 0.4	<0.02
Protein (%)	20 ± 0.5	15 ± 0.6	N.S
Cholesterol (g)	163 ± 13.1	103 ± 9.1	<0.001
Fiber (g)	14 ± 0.6	16 ± 0.8	<0.02

*Student's *t*-test for unpaired data.

## Abbreviations

BG: Blood glucose
CHO: Carbohydra te
HDL-C: High-density lipoprotein cholesterol
iAUC: Incremental area under the curve
LDL-C: Low-density lipoprotein cholesterol
MetS: Metabolic syndrome
MetS−: Patients without metabolic syndrome
MetS+: Patients with metabolic syndrome
TG: Triglycerides.
